# Interventions to Prevent Patient‐ and Visitor‐Perpetrated Violence Against Nurses in the Emergency Department: A Scoping Review

**DOI:** 10.1111/jan.70338

**Published:** 2025-11-03

**Authors:** Mariarosaria Gammone, Daniela Cattani, Martina Barbieri, Andrea Moro, Milko Zanini, Gianluca Catania, Loredana Sasso, Fiona Timmins, Annamaria Bagnasco

**Affiliations:** ^1^ Department of Health Sciences University of Genoa Genoa Italy; ^2^ School of Nursing, Midwifery and Health Systems, University College Dublin Dublin Ireland

**Keywords:** multiprofessional education, nurse ‐ patient interaction, violence

## Abstract

**Aim:**

To identify interventions and strategies to prevent patient‐ and visitor‐perpetrated violence against nurses working in acute hospital Emergency Departments. Design Scoping review following the Joanna Briggs Institute guidelines.

**Methods:**

A comprehensive literature search was conducted in PubMed and CINAHL to identify relevant studies published up to June 2024. The review included primary research studies employing quantitative, qualitative, and mixed‐method approaches. Eligibility criteria focused on interventions aimed at preventing physical and verbal violence in acute hospital emergency settings, explicitly targeting nurses. The selection process followed PRISMA‐ScR guidelines, with independent screening and data extraction by two reviewer pairs. Data Sources PubMed and CINAHL databases were searched for studies published up to June 2024.

**Results:**

A total of 40 studies were included, covering interventions across 11 countries, mostly from the United States. Interventions were categorised as organisational, environmental, or individual‐focused. Training programs were the most common strategy, followed by risk assessment tools, defense strategies, multidisciplinary briefings, and technology‐assisted interventions. Most interventions (73%) were implemented before violent incidents, 23% during, and 5% after. Healthcare workers, particularly nurses, were the primary target group, highlighting the need for effective preventive strategies.

**Conclusion:**

Violence prevention interventions in Emergency Departments focus on pre‐incident strategies, mainly organisational and individual‐focused. Limited attention has been given to environmental interventions despite their role in mitigating workplace violence. Further research is needed to assess the long‐term effectiveness of these strategies.

**Implications for the Profession and/or Patient Care:**

Addressing workplace violence in EDs ensures a safer work environment, improves staff retention, and enhances patient care quality.

**Patient or Public Contribution:**

This study did not include patient or public involvement in its design, conduct, or reporting.

## 1 Introduction

Among various professional sectors, healthcare stands out as one of the most exposed to the risk of workplace violence (WPV). In recent years, awareness of WPV affecting healthcare workers has significantly increased, partly due to the rising scale of the problem globally (Rossi et al. 2023). This situation is reflected in Italy as well, as demonstrated by the recent report published by the Italian Ministry of Health (2024). These data indicate that there are approximately 12 million reported assaults against healthcare workers. Indeed, there was a recent coverage in national newspapers that not only recounts violent episodes in hospitals but also discusses interventions implemented by healthcare organisations to address the issue (IlPost 2024a, 2024b). Recent estimates suggest that between 25% and 75% of healthcare workers have experienced WPV (Rossi et al. 2023).

Emergency and urgent care departments, as well as mental health units, are particularly vulnerable settings due to their inherent working conditions and patient population characteristics (Bagnasco et al. 2024; McLaughlin and Khemthong 2024; Rossi et al. 2023). Moreover, in the literature, nurses are identified as the professional group at the highest risk of experiencing WPV in the healthcare sector (Doehring et al. 2024; Tiesman et al. 2023).

WPV encompasses a wide range of acts, from verbal threats and harassment to physical assaults. The National Institute for Occupational Safety and Health (NIOSH) defines WPV as violent acts, including physical assaults and threats, directed at individuals who are at work or on duty (National Institute for Occupational Safety and Health 2002). Moreover, the World Health Organization (WHO) has identified the prevention of all forms of violence as a fundamental public health priority, emphasising the need for further research on this critical issue (World Health Organization 2002). A recent systematic review (Rossi et al. 2023) indicated that verbal assaults are the most common form, followed by physical violence. Additionally, this review highlighted key contributing factors, including prolonged waiting times, staff shortages, unrestricted public access to facilities, and patient conditions such as substance abuse or psychiatric disorders. WPV shares common characteristics across different cultural contexts.

A critical issue highlighted in the literature is the underreporting of violent incidents. This reluctance to report is often linked to organisational factors, including perceptions of inadequate support and the belief that reporting is ineffective. Furthermore, healthcare workers frequently perceive violence, particularly verbal abuse, as an inherent part of their job (Rossi et al. 2023; Tyler et al. 2022). Understanding the root causes of WPV is essential for developing effective preventive strategies. Al‐Qadi (2021) categorises antecedent factors into three main groups: external factors (such as policies and work environment), perpetrator‐related factors, and victim characteristics. These findings align with the multidimensional framework proposed by Ramacciati et al. (2018), which underscores the complex interplay between individual, environmental, and organisational factors.

The consequences of WPV are far‐reaching, affecting healthcare workers on multiple levels. Physically, WPV can result in injuries from assaults. Psychologically, it contributes to anxiety, depression, post‐traumatic stress disorder, and other mental health conditions (Leźnicka and Zielińska‐Więczkowska 2024; Arnetz et al. 2025). WPV is also a significant predictor of burnout, exacerbating its negative effects and leading to decreased job satisfaction and an increased intention to leave the profession (Vidal‐Alves et al. 2022).

This scoping review aims to explore the strategies currently employed to prevent violence against healthcare workers in emergency departments, emphasising evidence‐based interventions and highlighting persistent organisational and cultural challenges.

## 2 Aim

The aim of this study is to identify interventions or strategies to prevent patients and/or visitors from perpetrating violence against healthcare workers in acute hospital Emergency Departments (ED).

## 3 Methods

### 3.1 Design

The study is a scoping review performed according to the JBI guidelines (Peters et al. 2020). The research question aligned with the study aim, which guided the setting of the study was: what are the interventions or strategies to prevent patient‐ and/or visitor‐perpetrated violence against nurses in acute hospital Emergency Department settings?

### 3.2 Search Methods

Considering our study objective, the Population, Exposure and Outcome (PEO) strategy was employed to identify relevant keywords for our research and to facilitate the construction of the search string, as shown in Table 1.

**TABLE 1:** PEO strategy, keywords and search strategy.

	Keywords	Search strategy
Population	Hospital Emergency nurse	Nurs* AND (Emergency department OR emergency unit OR emergency room OR emergency service, hospital [MeSH]) AND Hospital
Exposure	ED patient perpetrated violence ED attendee perpetrated violence ED visitor perpetrated violence ED accompaniment perpetrated violence Violence Physical violence Verbal violence Violent behaviour Aggression Attack Aggression Assault Threat	(violen*) OR (aggress*) OR (attack*) OR (assault*) OR (threat*) OR (“violent behaviour*”)
Outcome	Approach to reduce violence De‐escalation strategies Intervention Education Policy Security measure Management	Training* OR Education* OR Intervention* OR environment measure* OR Behaviour* OR Organisational polic* OR Security measure* OR strateg* OR approach* OR policy OR manag* OR prevent*OR Approach* OR Avoidance* OR Debriefing OR De‐escalation OR Deterrence OR Education* OR Policy OR Zero‐tolerance policy OR Program OR Provision OR Reduction OR Provide OR support

**FIGURE 1:**
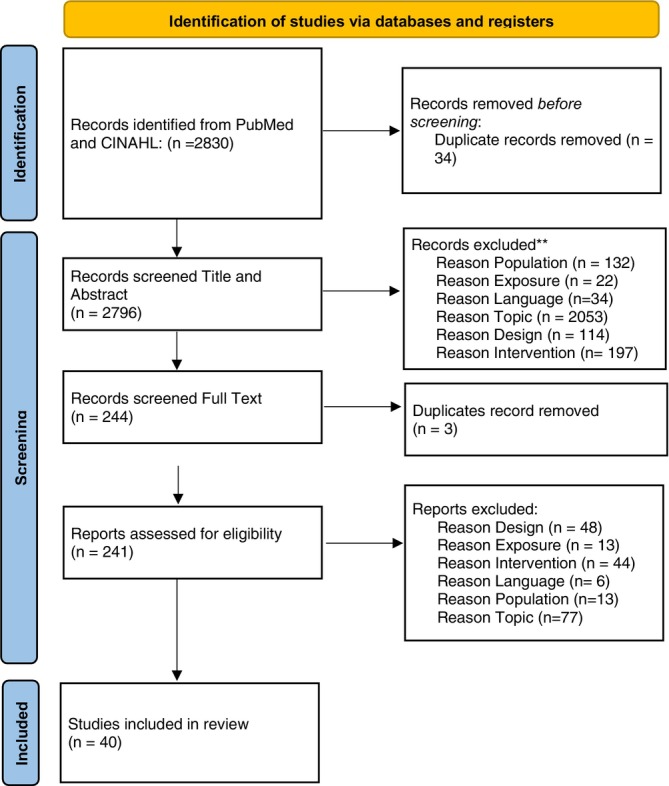
PRISMA 2020 flow diagram. This work is licensed under CC BY 4.0. To view a copy of this licence, visit https://creativecommons.org/licenses/by/4.0/. *Source:* Page et al. 2021. BMJ 372: n71. https://doi.org/10.1136/bmj.n71.

A comprehensive literature search was conducted using PubMed and CINAHL to identify relevant studies published up to June 2024. In line with PRISMA recommendations (Tricco et al. 2018), the study selection process was conducted in two stages. First, two pairs of independent reviewers screened the titles and abstracts of identified studies to assess their relevance. This was followed by a full‐text review, where the same pairs of reviewers evaluated the selected articles for eligibility. Any disagreements that arose during the review process were resolved collaboratively, with each pair addressing the uncertainties of the other group to reach a consensus. All the screening processes have been represented using the PRISMA diagram (Figure 1).

In accordance with the scoping review methodology proposed by Arksey and O'Malley (2005), and refined by Levac et al. (2010) and the Joanna Briggs Institute (Peters et al. 2020), we did not conduct a formal critical appraisal of the included sources. This decision aligns with the aim of the present review, which was to provide a comprehensive mapping of the available literature, irrespective of study quality, to identify key themes and gaps in knowledge. As such, the inclusion of studies was based on relevance to the research question rather than methodological rigour. While critical appraisal is mentioned in the PRISMA‐ScR checklist, it is not a mandatory component of scoping review methodology.

### 3.3 Inclusion and Exclusion Criteria

This review focuses on studies that investigate interventions aimed at preventing physical and verbal violence, as well as aggression, within emergency healthcare settings. Specifically, it includes research conducted in acute hospital adult EDs or in settings where interventions explicitly targeted the ED environment. To ensure a comprehensive analysis, only original primary research studies were considered, encompassing quantitative, qualitative, and mixed‐method approaches. A key inclusion criterion was the explicit identification of perpetrators as patients, visitors, caregivers, escorts, or accompaniments, which allows for a clearer understanding of the sources of violence within EDs. Furthermore, only studies published in English were included to maintain consistency in language accessibility and comparability. Particular attention was given to studies that specifically targeted the prevention and management of violence against healthcare workers working in EDs.

### 3.4 Data Abstraction

The included records were analysed independently by the two groups, who extracted the relevant data to address the study's aim and to feature the interventions identified according to the Cochrane Review by Spelten et al. (2020), indicating whether the intervention is meant to be implemented before, during, or after the violent incident, and if it directly affects victims, perpetrators, or the care settings. Moreover, according to the WHO guidelines (2002) about WPV, the data extraction involved categorising the interventions as organisational, environmental, or individual‐focused.

## 4 Results

To address the research question, the included studies were synthesised based on their key characteristics. A total of 40 interventions aimed at preventing violence in ED settings were identified. These studies were conducted across 11 countries, with the majority originating from the United States. Notable contributions also included five studies from the United Kingdom, three each from Iran and Australia, and smaller contributions from Italy, Canada, and other regions, each represented by one or two studies. The general information of the included studies is summarised in Table 2.

**TABLE 2:** Paper information, details about the interventions.

Paper information				Intervention		Timing of the intervention	Focus of the intervention on	Type of intervention
First author's name	Year	Country	Design	Characteristics	Efficacy	Before the violent incident/during the violent incident/after the violent incident	The Healthcare workers/victims patients/perpetrators the care setting	Organisational interventions, environmental interventions, individual‐focussed interventions
Chang Y. C. et al.	2022	Switzerland	Experimental/Quasi‐experimental	Training Program	Descriptive and inferential statistics	Before the violent incident	The Healthcare workers/victims	Individual‐focussed interventions
D'Ettorre G. et al.	2020	Italy	Other	Risk Assessment Tool	Descriptive and inferential statistics	Before the violent incident	The care setting	Organisational interventions
Bruccoli AM	2023	United States	Quality improvement	Multidisciplinary Breifing	Descriptive and inferential statistics	Before the violent incident	The Healthcare workers/victims	Organisational interventions
Kotora J.G. et al.	2014	United States	Experimental/Quasi‐experimental	Training Program	Descriptive and inferential statistics	Before the violent incident	The Healthcare workers/victims	Individual‐focussed interventions
Fernandes C.M. et al.	2002	United States	Observational Study	Training Program	Descriptive and inferential statistics	Before the violent incident	The Healthcare workers/victims	Individual‐focussed interventions
Sharifi S. et al.	2020	England	Experimental/Quasi‐experimental	Training Program	Descriptive and inferential statistics	Before the violent incident	The Healthcare workers/victims	Individual‐focussed interventions
Gillespie G.L. et al.	2014	USA	Experimental/Quasi‐experimental	Training Program	Descriptive and inferential statistics	Before the violent incident	The Healthcare workers/victims	Organisational interventions
Deans C.	2004	Australia	Experimental/Quasi‐experimental	Training Program	Descriptive and inferential statistics	Before the violent incident	The Healthcare workers/victims	Individual‐focussed interventions
Gerdtz M.F. et al.	2013	England	Mixed‐Method Study	Training Program	Descriptive and inferential statistics	Before the violent incident	The Healthcare workers/victims	Individual‐focussed interventions
Gillam S.W.	2014	United States	Observational Study	Training Program	Descriptive and inferential statistics	Before the violent incident	The Healthcare workers/victims	Individual‐focussed interventions
Gramling J.J. et al.	2018	United States	Observational study	Defence Strategy	Descriptive and inferential statistics	During the violent incident	The care setting	Organisational interventions
Larson L.A. et al.	2019	Netherlands	Experimental/Quasi‐experimental	Risk Assessment Tool	Descriptive and inferential statistics	During the violent incident	The Healthcare workers/victims	Environmental interventions
Cabilan C.J. et al.	2023	United States	Observational Study	Risk Assessment Tool	Descriptive and inferential statistics	Before the violent incident	Patients/perpetrators	Organisational interventions
Wong A.H. et al.	2015	United States	Experimental/Quasi‐experimental	Training Program	Descriptive and inferential statistics	Before the violent incident	The Healthcare workers/victims	Organisational interventions
Daniel C. et al.	2015	England	Observational Study	Risk Assessment Tool	Descriptive and inferential statistics	Before the violent incident	The Healthcare workers/victims	Individual‐focussed interventions
Senz A. et al.	2021	Australia	Observational study	Risk Assessment Tool	Descriptive and inferential statistics	Before the violent incident	The Healthcare workers/victims	Organisational interventions
Dickinson T. et al.	2020	England	Observational Study	Defence Strategy	No Evidence	During the violent incident	Patients/perpetrators	Individual‐focussed interventions
Quinn J.M. and Koopman J.M.	2023	United States	Observational study	Risk Assessment Tool	Descriptive and inferential statistics	Before the violent incident	Patients/perpetrators	Organisational interventions
Lee H. et al.	2023	Korea	Observational Study	Technology assisted strategy	Descriptive and inferential statistics	After the violent incident	The care setting	Organisational interventions
Carr M.A. and Derouin A.	2023	United States	Mixed‐Method Study	Defence Strategy	No Evidence	During the violent incident	The care setting	Environmental interventions
Krull W. et al.	2019	United States	Quality improvement	Training Program	Descriptive and inferential statistics	Before the violent incident	The Healthcare workers/victims	Individual‐focussed interventions
Luck L. et al.	2007	England	Qualitative study	Risk Assessment Tool	No Evidence	Before the violent incident	The Healthcare workers/victims	Environmental interventions
Gillespie G.L. et al.	2013	United States	Other	Training Program	Descriptive and inferential statistics	Before the violent incident	The care setting	Organisational interventions
Quinn, Janis M. and Koopman, Joy M.	2023	USA	Observational Study	Risk Assessment Tool	Descriptive and inferential statistics	During the violent incident	The Healthcare workers/victims	Organisational interventions
Connor M. et al.	2020	United States	Observational study	Risk Assessment Tool	Descriptive and inferential statistics	Before the violent incident	Patients/perpetrators	Organisational interventions
Cabilan, C.J. et al.	2022	Australia	Mixed‐Method Study	Risk Assessment Tool	Descriptive and inferential statistics	Before the violent incident	The care setting	Organisational interventions
Campbell E. et al.	2021	USA	Observational Study	Risk Assessment Tool	Descriptive and inferential statistics	During the violent incident	The Healthcare workers/victims	Organisational interventions
Sharifi S. et al.	2020	Iran	Experimental/Quasi‐experimental	Training Program	Descriptive and inferential statistics	During the violent incident	Patients/perpetrators	Environmental interventions
Movaghari Sadatmahaleh M. et al.	2019	Iran	Experimental/Quasi‐experimental	Training Program	Descriptive and inferential statistics	Before the violent incident	The Healthcare workers/victims	Organisational interventions
Wu J.‐C. et al.	2019	Taiwan	Observational Study	Training Program	Descriptive and inferential statistics	Before the violent incident	The Healthcare workers/victims	Individual‐focussed interventions
Phillips S	2007	United States	Observational Study	Training Program	No Evidence	Before the violent incident	The Healthcare workers/victims	
Gillam S.W.	2014	United States	Observational Study	Training Program	Descriptive and inferential statistics	During the violent incident	The Healthcare workers/victims	Individual‐focussed interventions
Cahill D	2008	United States	Experimental/Quasi‐experimental	Training Program	Descriptive and inferential statistics	Before the violent incident	The Healthcare workers/victims	Individual‐focussed interventions
Burkoski V et al.	2019	Canada	Qualitative study	Technology assisted strategy	No Evidence	After the violent incident	The Healthcare workers/victims	Environmental interventions
Smith MA	2023	United States	Quality improvement	Multidisciplinary Briefing	Descriptive and inferential statistics	Before the violent incident	The care setting	Organisational interventions
Hemati E. et al.	2018	Iran	Other	Training Program	Descriptive and inferential statistics	Before the violent incident	The Healthcare workers/victims	Individual‐focussed interventions
Eslamian J et al.	2010	India	Experimental/Quasi‐experimental	Training Program	Descriptive and inferential statistics	Before the violent incident	The Healthcare workers/victims	Individual‐focussed interventions
Brown RG et al.	2018	United States	Observational Study	Training Program	Descriptive and inferential statistics	During the violent incident	The care setting	Individual‐focussed interventions
Kim S.C. et al.	2022	United States	Observational Study	Risk Assessment Tool	Descriptive and inferential statistics	Before the violent incident	The care setting	Organisational interventions
Mallett‐Smith S et al.	2023	United States	Quality improvement	Violence prevention Bundle—Risk Assessment Tool, Training Program, Defence Strategy	Descriptive and inferential statistics	Before the violent incident	The Healthcare workers/victims	Organisational interventions

The studies spanned from 2002 to 2023, with a notable concentration in the most recent year. In terms of study design, 16 studies (40%) were observational, 11 (28%) experimental or quasi‐experimental, 5 (13%) quality improvement projects, 3 (8%) mixed‐method studies, 2 (5%) qualitative studies, and 3 (8%) employed other designs.

All the papers included reported intervention, but evidence of efficacy was reported only by 34 studies (85%) through descriptive and inferential statistics, while eight studies (20%) indicated insufficient or no evidence of effectiveness. The interventions were categorised based on their characteristics. Simulation‐training programs were the most frequently reported strategy, implemented in 20 studies (e.g., Chang et al. 2022; Kotora et al. 2014; Fernandes et al. 2002; Sharifi et al. 2020; Gillespie et al. 2014). Risk assessment tools were utilised in 13 studies (e.g., D'Ettorre et al. 2020; Larson et al. 2019; Cabilan et al. 2023; Quinn and Koopman 2023). Defence strategies were highlighted in three studies (Gramling et al. 2018; Dickinson and Clark 2020; Carr and Derouin 2023), multidisciplinary briefings in two studies (Bruccoli 2023; Smith 2023), and technology‐assisted strategies in two studies (Lee et al. 2023; Burkoski et al. 2019). A single study (Mallett‐Smith et al. 2023) proposed a comprehensive violence prevention bundle combining risk assessment tools, simulation‐training programs, and defence strategies. Regarding timing, 75% of the interventions (thirty studies) were designed to be implemented before violent incidents occurred, 25% (ten studies) during violent incidents, and 5% (two studies) after violent incidents (Table 2).

The primary focus of 28 interventions was on healthcare workers or victims, emphasising the protection of healthcare staff (Table 2). Nine interventions targeted the ED care setting by modifying environmental factors to mitigate violence risks, while five interventions focused on patients or perpetrators. The interventions were further categorised by type: organisational interventions accounted for 48% (19 studies), individual‐focused interventions for 43% (seventeen studies), and environmental interventions for 15% (six studies).

Table 3 provides an overview of these categories, along with an outline of the type of violence prevention interventions. Among those targeting patients or perpetrators, most were implemented before the violent incidents (3 out of 5) and were primarily organisational strategies. During violent incidents, interventions included one environmental, and one individual‐focused strategy.

**TABLE 3:** Numerosity of interventions according to category, timing, and target.

Category	Timing	Organisational	Environmental	Individual‐focused
Patients/Perpetrators	Before	3	0	0
	During	0	1	1
	After	0	0	0
The care setting	Before	5	0	0
	During	1	1	1
	After	1	0	0
Healthcare workers/Victims	Before	6	2	13
	During	2	1	1
	After	0	1	0

*Note:* Category: refers to the population/setting to whom the preventive interventions are addressed. Timing: refers to the point at which the intervention is implemented in relation to a violent event. Organisational, Environmental, Individual‐focused: This indicates the type of prevention strategy, specifying whether the intervention is organisational, environmental, or person‐focused.

Interventions targeting the care setting were predominantly designed for implementation before violent incidents (5 out of 9) and primarily employed organisational strategies (Table 3) During violent incidents, one intervention in each category (organisational, environmental, and individual‐focused) was identified, while only one organisational intervention addressed violence after incidents occurred. For interventions targeting healthcare workers or victims, a substantial majority (21 out of 26) were implemented before violent incidents. Of these, 13 were individual‐focused, 6 were organisational, and 2 were environmental strategies. During incidents, 4 interventions were identified, including 2 organisational, 1 environmental, and 1 individual‐focused approach. Only one intervention, an environmental strategy, was specifically designed to address violence after it occurred. These findings underscore that most interventions aim to prevent violence before its occurrence, particularly through organisational and individual‐focused strategies. Healthcare workers or victims represent the primary target group, highlighting a critical focus on protecting frontline staff in ED settings.

## 5 Discussion

This scoping review aimed to identify the most effective interventions used in ED to prevent WPV against healthcare workers (Table 4). The number of included studies suggests that several interventions could be planned to prevent aggressions. Among these, simulation‐based training programs may be the most effective in improving healthcare workers' ability to recognise and manage WPV.

**TABLE 4:** Summary of included studies by intervention description, sample characteristics, evaluation method, and evidence of efficacy.

First author's name	Year	Descrizione dell'intervento	Participant (type/N)	How efficacy was measured	Efficacy (Yes/No/Not testes)
Chang Y. C. et al.	2022	The Workplace Violence Prevention and Management Training Program (WPV‐PMTP) involved randomly assigning participants to two groups, each attending different training sessions. Outcomes were evaluated based on a theoretical framework using various assessment tools, including measures of demographics, goal commitment, occupational coping self‐efficacy, attitudes toward aggression in the emergency room, and confidence in managing aggressive behaviour	Emergency Department Staff (N. 75)	The intervention significantly improved goal commitment, coping self‐efficacy, confidence in handling violence, and attitudes toward PVV among ED nurses. Model fit was strong across outcomes (marginal R^2^ = 0.29–0.72, *p* < 0.05)	Yes
D'Ettorre G et al.	2020	21 items‐Questionnaire (EDWPV‐Q) to calculate the risk of type II WPV were both Organisational and Environmental factors were considered	Expert to validate the tool (N.12)	The tool was only validated by expert (Cronbach's alpha 0.90)	Not tested
Bruccoli AM et al.	2023	Specific protocol that involved three ED workers in rotation (ED RN, a social worker, and a security member) that do a pre‐intervention briefing on a patient. Post‐intervention survey with a electronic debriefing tool in which was recorded the time, another post‐debriefing tool were filled in by a research team	Emergency Department Staff (N. 43 pre‐implementation/N.21 post‐implementation)	The perception of safety was higher in post‐implementation period mean 2.95 (SD = 0.86, 95% CI [0.66, 1.25]). In the OWPV wasn't observed any significant differences in post‐implementation period	Yes
Kotora J.G. et al.	2014	Simulation‐based disaster training program for ED staff conducted at a simulation centre. Participants completed a pre‐test 10 items‐questionnaire, were then divided into equal multidisciplinary teams, and took part in a simulated disaster exercise. A post‐test questionnaire was administered at the end of the session to assess outcomes	ED medical students, physician, nurses (N.32)	There was a statistically significant improvement in the average scores from the pre‐test to the post‐test questionnaire (*p* < 0.002; 95% CI: −0.177 to −0.041). Paired Student's t‐tests confirmed this difference across all participants, indicating a significant effect of the intervention (*p* < 0.002; 95% CI: −0.177 to −0.041)	Yes
Fernandes C.M. et al.	2002	The intervention was a prevention and Management of Aggressive Behaviour Program to assess and prevent aggressive behaviour and consisted in a 4‐h course for a group of max 40 people. To measure the program efficacy, they used a Surevey before and after the intervention (3 months and 6 months after)	ED Staff: 667 of 798 surveys were completed (84%)	A significant reduction in both physical and verbal violence was observed at 3 months post‐intervention. Physical violence incidents dropped from 49 to 19 (OR 0.35; 95% CI: 0.15–0.84), and verbal violence from 154 to 58 (OR 0.31; 95% CI: 0.21–0.46). At 6 months, the reduction in verbal violence remained significant (OR 0.47; 95% CI: 0.33–0.66), while physical violence slightly increased but remained below baseline (OR 0.79; 95% CI: 0.48–1.40)	Yes
Sharifi S. et al.	2020	Participants attended a workshop focused on a violence risk assessment checklist and a preventive protocol. The checklist was completed during patient admissions, and the protocol was implemented according to the identified risk level. Effectiveness was evaluated through pre‐ and post‐intervention surveys	ED Nurses (N.37)	The intervention proved highly effective, with a significant reduction in overall violence scores (from 8.4 to 2.7; *p* < 0.0001). Statistically significant improvements were also observed in the frequency of verbal abuse (*p* < 0.0001), perception of workplace security (*p* = 0.006), fear of injury (*p* < 0.02), and response to violent incidents (*p* < 0.01)	Yes
Gillespie G.L. et al.	2014	The intervention consisted of environmental modifications, new workplace violence (WPV) policies and procedures, and a comprehensive education and training program. It was developed collaboratively with hospital staff and implemented over three months in emergency departments, with ongoing support and monitoring by the research team	ED Staff (N. 209)	No statistically significant differences in assault rates were observed between the two groups. However, two intervention sites reported a reduction in the incidence of assaults and physical threats	No
Deans C. et al.	2004	The intervention consisted of a one‐day training program. Questionnaires were administered two months prior to the training, and a post‐test questionnaire was completed three months after the training had concluded	ED Nurses (N.40)	Significant improvements were observed in participants' knowledge (*p* = 0.001), skills (*p* = 0.006), and awareness of personal and others' physical limitations (*p* ≤ 0.05). Aggressive incidents decreased post‐intervention, though not significantly (*p* = 0.06). No significant changes in team response or duty of care	Yes
Gerdtz M.F. et al.	2013	The program is a structured, evidence‐informed 45‐min training session designed to prevent and manage patient aggression in emergency departments. It includes a video simulation, discussion of research evidence, and a guided reflection on current practices to promote safer and more effective responses to aggression	ED Nurses (N.913 took part in the program, N.755 responded in the survey)	Efficacy was assessed using the Management of Aggression and Violence Attitude Scale (MAVAS) before and 6–8 weeks after training, with 471 matched responses. The intervention had limited effectiveness. Significant attitude changes occurred in 4 of 12 items related to causes of aggression and 1 of 11 items on management. Participants remained uncertain about aggression prevention and restraint	No
Gillam S.W.	2014	An 8‐h Nonviolent Crisis Intervention program for Emergency Department staff, designed to enhance their ability to de‐escalate potentially violent situations	ED Staff (N. 104)	NCI training was associated with a 23% reduction in code purple incidents. A negative correlation was found between recent training (within 90–150 days) and violence rates, indicating that ongoing training effectively lowers aggression in the ED	Yes
Gramling J.J. et al.	2018	The introduction of conducted electrical weapons (CEW) carriage by hospital security officers	Study personnel (N 1034), of whom N 468 nurses	Poisson‐like regression compared injury rates before/after CEW use. Despite no significant reduction in injury rates (RR = 1.0, 95% CI 0.7–1.4), injury costs among security staff decreased post‐implementation, suggesting reduced injury severity	No
Larson L.A. et al.	2019	Quality and improvememt team developed a tool whic they tested using the methodology of Plan‐Do‐Check‐Act. As part of the intervention, an ED nurse initiated a structured huddle by notifying the receiving unit of patients identified as at risk for violence, followed by a joint handoff call with the admitting team to facilitate a coordinated transition	NA	This protocol was applied to 21 transfers during the first PDSA cycle and 18 in the second. Following implementation, 100% of RNs across the ED and six medical units reported feeling safe during transfers (vs. 54.7% at baseline), and ED nurse satisfaction with handoffs increased from 53.3% to 75.0% between the first and second cycles	Yes
Cabilan C.J. et al.	2023	The intervention consisted of the introduction of a tool for patient risk assessment, which collected data on three risk factors: history of aggression, behavioural concerns, and clinical presentation concerns; patients were then classified into three risk categories. As part of the implementation, staff received training on the tool via an e‐learning platform	ED Nurses (N.149)	Training completion: 76% of nurses; total usage: 65% of patient in the ED were assessed with the tool. There was a reduction in number of reported incidents compared to baseline	Yes
Wong A.H. et al.	2015	Immersive simulated encounters involving two distinct case studies were used to evaluate the effectiveness of a three‐hour training session aimed at improving healthcare professionals' violence prevention skills. All participants completed pre‐ and post‐training surveys to assess changes in knowledge and confidence. A total of ten training sessions were conducted, with all healthcare professionals attending one session each	ED staff (N.162)	Significant improvements were observed in perceptions of internal, external, and situational factors related to patient aggression (*p* < 0.0001, *p* < 0.002, *p* < 0.0001), though attitudes toward aggression management did not change significantly (*p* = 0.542). Several quality improvement initiatives were implemented, and staff expressed positive feedback on the simulation‐based training and interaction with standardised patients	Yes
Daniel C. et al.	2015	A violence risk‐screening process at ED entry to identify patients at risk of aggression and guide targeted management strategies	ED triage nurses (N.9) that conducted 167 triage assessment	No intervention effectiveness was measured. The study used observations and interviews to assess how violence risk is currently screened and whether the public supports a structured approach. Findings suggest that a standardised screening process is needed but not consistently applied in practice	Not tested
Senz A. et al.	2021	The intervention involved integrating specific violence checklist, a tool predicting violence risk within 24 h, into the ED nursing observation chart. Based on scores from 0 to 6, an embedded response matrix guided multidisciplinary actions such as de‐escalation, medication, or restraint	ED Nurses: pre‐implementation (N.76); post‐implementation (N.83)	The intervention resulted in a significant reduction in unplanned security responses to workplace violence (RR = 0.75) and an increase in planned security interventions (RR = 2.22). Staff confidence in violence risk assessment rose from 82% to 91%. Documented risk assessments increased from 30% pre‐intervention to 82% post‐intervention (*P* < 0.0001)	Yes
Quinn J.M. et al.	2023	Implementation of a specific violence checklist scoring by nurses for ED patients to track the reporting of violence incidence	N 68 individual assessment on a total of N 27 patients	Effectiveness was assessed by comparing the number of violent incidents identified using specific Checklist versus those reported in the hospital system, and by monitoring changes in scores after interventions. There was an increasing in reporting incident (from a score of 3.3 incidents per month to15.1 per month)	Yes
Lee H. et al.	2023	The intervention involved developing predictive models for workplace violence in the Emergency Department. Data were extracted from the clinical data warehouse, with cases of violence identified through nursing documentation using the International Classification of Nursing Practice (ICNP) terminology related to violent incidents	Data from 7259 patients were utilised in the analysis	Random Forest achieved the highest predictive performance, with an accuracy of 0.90 (95% CI: 0.898–0.912) when both ED visit and stay factors were used	Yes
Krull W. et al.	2019	Interprofessional staff training using computer‐based modules and simulations on de‐escalation and restraint techniques to enhance skills when working with violent patients	Simulation participants (N 96), of whom N 52 registered nurses	Effectiveness was evaluated through self‐assessments of knowledge, skills, abilities, confidence, and preparedness conducted before and after the training. Results showed significant improvements, with preparedness increasing by 30% (*P* < 0.0001) and knowledge by 21% (*p* < 0.0001). Additionally, satisfaction levels were higher among less experienced staff (*p* = 0.029)	Yes
Gillespie G.L. et al.	2013	The multifactorial intervention included customised workplace violence (WPV) policies and procedures, standardised interprofessional online training on violence prevention and management for staff, and enhancements to environmental safety measures. Participants evaluated the program using a Likert scale	ED Staff (N.80): N. 35 ED nurses	They conducted descriptive statistics without directly assessing effectiveness. Results showed mixed success in implementation. Nurses rated the program as more beneficial compared to physicians, who had low participation	Not tested
Quinn J.M. et al.	2023	Following completion of the educational course on the project, nurses in the adult emergency department began implementing the Broset Violence Checklist. The checklist was provided at each nurse's workstation, and nurses were instructed to complete it for every patient displaying any of the behaviours specified in the tool	ED nurses (N.65)	The Broset Violence Checklist captured significantly more incidents than the standard hospital reporting system. Higher checklist scores were significantly associated with interventions such as providing comfort measures, addressing concerns, and applying restraints. The Broset Violence Checklist was effectively utilised in the emergency department to identify behaviours predictive of violence	Yes
Connor M. et al.	2020	The intervention consist in the use of the Dynamic Appraisal of Situational Aggression (DASA), an assessment tool used to predict violent or aggressive behaviour	3433 scores representing 1584 patients	The instrument was evaluated in an emergency setting and demonstrated predictive validity for assessing behavioural health patients in an urban academic emergency department	Not tested
Cabilan C.J. et al.	2022	A brief screening tool assessing risk of occupational violence across three domains (aggression history, behavioural concerns, and clinical presentations). Each domain is scored 0 (no) or 1 (yes); total scores (0–3) indicate low (0), moderate (1), or high (2–3) risk	ED staff (NA)	The tool was tested for Content validity (*N* = 81), predictive validity, inter‐rater reliability. AUC = 0.77 (95% CI: 0.7–0.81); moderate risk: 61% sensitivity, 91% specificity; high risk: 37% sensitivity, 97% specificity. Cohen's kappa = 0.67–0.75. Acceptable validity and moderate inter‐rater agreement support use in EDs	Not tested
Campbell E. et al.	2021	The Emergent Documentation Aggression Rating Tool to help ED nurses identify, document, and respond to escalating patient behaviour	32,290 unique ED patients (48,492 visits)	Chi‐square test, logistic interrupted time series, chart audits, nurse surveys were performed to evaluate efficacy of the intervention. EDART use significantly increased (χ^2^(1) = 260.8, *p* < 0.001); restraint use decreased (0.11% to 0.07%) but not statistically significant (*p* = 0.13); de‐escalation at discharge rose from 1.3% to 5.8%	Yes
Sharifi S. et al.	2020	TRAINING INTERVENTION (Workshop): A 6‐week workshop for nurses focused on instruction in the use of a risk assessment checklist (SOAS‐R) and implementation of a preventive protocol	ED nurses (n. 37)	The mean SOAS‐R violence severity score significantly decreased from 8.4 ± 4.5 pre‐intervention to 2.7 ± 3.9 post‐intervention (*p* < 0.001). Similarly, verbal abuse incidents dropped from 2.9 ± 3.2 to 0.8 ± 1.8 (*p* = 0.0001). Before the intervention, nurses primarily reacted by defending themselves (37.5%), whereas after the intervention, most used multiple reaction types (35.74%) (*p* = 0.01)	Yes
Movaghari Sadatmahaleh M. et al.	2019	Two‐day workplace violence prevention training for ED nurses combined with environmental safety improvements (lighting, ventilation, CCTV, security guard)	ED nurses (n. 48)	Chi‐square test and McNemar's test comparing violence frequency/types before and after intervention in intervention and control groups. No significant difference in frequency of violence before vs. after intervention in intervention and control groups (*p* > 0.05, McNemar's test). Significant difference found in forms of violence before and one month after intervention within the intervention group (*p* = 0.004). Significant difference in place of psychological violence between groups (*p* < 0.001). Racial violence difference before intervention (*p* = 0.034), but none reported after. The intervention reduced certain forms of violence (significant change in forms of violence in intervention group), but overall frequency differences before and after were not statistically significant	No
Wu J.‐C. et al.	2019	The intervention consisted of a simulation course focused on emergency room violence. The authors used a pre‐post‐test design to evaluate the study. Outcomes included self‐efficacy assessments and trained evaluators' ratings of healthcare providers' responses during simulated workplace violence scenarios	ED staff (N. 34): N. 20 registered nurses	The simulation intervention was evaluated using pre‐, post‐, and delayed post‐tests. Significant improvements were observed in healthcare personnel's self‐efficacy and response to ER violence when comparing pre‐test to post‐test scores (Wald χ^2^ = 85.202, *p* < 0.001), while no significant differences were found between post‐test and delayed post‐test results	Yes
Cahill D. et al.	2008	The intervention consisted of an 8‐h educational training program based on the Assertive Communication Techniques of the Scripps Mercy Aggression Reduction Training (ACT‐SMART). Data were collected using multiple tools before and after the training	ED staff (N. 65): 56 intervention group (N. 12 ED nurses) and N. 9 control group (ED nurses)	After attending ACT‐SMART, the experimental group (*n* = 56) showed significant improvements in confidence managing aggressive situations (*p* = 0.001 and *p* = 0.007). The control group's mean scores remained unchanged (2.4). Weekly verbal aggression exposure was reported by 25%–33% of participants	Yes
Smith M.A. et al.	2023	A behaviour management consultation team led by Clinical Nurse Leader (CNL) was established to support clinical staff in handling aggressive or violent patients. The team conducted consultations to identify underlying causes of agitation and implemented targeted strategies, including safety reminders and behavioural contracts	ED staff: NA Behaviour management consultants: N.108	The authors did not assess the efficacy of the intervention, but instead evaluated ED staff satisfaction through a survey. The majority of respondents reported being satisfied with the consultation	No
Hemati‐Esmaeili M. et al.	2018	The intervention consisted in a workplace violence prevention program (WVPP) aimed at reducing violence against nurses perpetrated by patients and their families	ED nurses (N.49)	WVPP significantly reduced verbal violence (*p* = 0.007), mobbing/bullying (*p* = 0.001), fear of violence (lower mean from 46.1 to 34.3; *p* < 0.001), and concern about violence risk (lower mean from 3.4 to 3.0; *p* = 0.006). No change in physical violence (*p* = 0.754). Post‐intervention, more nurses saw verbal violence as uncommon (76%) and avoidable (56%), though reporting remained low and follow‐up often unsatisfactory. No significant associations were found with background variables	Yes
Eslamian J et al.	2010	The intervention consisted in an anger management training program	ED nurses (N.66)	The study employed chi‐square and Fisher's exact tests, as well as frequency distributions, to analyse occurrences of physical violence against nurses in the intervention group. A significant difference in the frequency of psychological violence was observed after the intervention, as indicated by the chi‐square test (*p* ≤ 0.04). Additionally, an independent t‐test (*p* = 0.001) revealed a significant difference in the mean anger management score before the intervention (59.52 ± 9.12). A paired t‐test further demonstrated a significant improvement in anger management scores following the intervention within the test group (19.5 ± 8.4)	No
Brown R.G. et al.	2018	The intervention consisted of a four‐hour workplace violence training program that integrated classroom instruction, simulation exercises, and practical self‐defence techniques	ED nurses (pre‐test N.196, post‐test N.136)	The intervention group demonstrated a significant increase in confidence when managing violent incidents (*p* < 0.05)	Yes
Kim S.C. et al.	2022	The intervention consisted of the routine completion of an expanded Aggressive Behaviour Risk Assessment Tool (ABRAT) by triage nurses for all walk‐in patients, followed by a violence checklist completed by the assigned nurse at patient discharge. The checklist included predefined categories of violence and an optional free‐text field to capture additional context	ED nurses of three different hospital (NA) 10,554 visitors	The authors assessed effectiveness through ROC analysis, finding a high discriminative ability (AUC = 0.91), with a cut off score of 1 yielding 84.3% sensitivity and 95.3% specificity, indicating that the ABRAT for EDs is effective in distinguishing violent from non‐violent patients	Yes
Mallett‐Smith S. et al.	2023	A violence prevention bundle was implemented, integrating a risk screening tool, an alert system, and targeted assault reduction strategies. This bundle was introduced alongside other ongoing workplace violence management initiatives	ED staff (NA)	The authors evaluated the effectiveness of the intervention by measuring the number of physical and verbal assaults during the post‐implementation period, observing a reduction in physical assaults	No tested

Simulations are effective because they mirror real‐life high‐stress situations and allow healthcare workers to practice de‐escalation techniques and emergency responses (Duncan et al. 2021). A crucial component of simulation training is the post‐simulation debriefing, which provides an opportunity for participants to reflect on their actions and decisions (Cabilan et al. 2022). The debriefing phase is especially important for adult learning, as it facilitates reflective practice and encourages a deeper understanding of the emotional and psychological dynamics at play during violent incidents (Brown et al. 2018). Reflective learning is fundamental to improving skills and promoting professional growth, especially in high‐risk fields such as healthcare where exposure to workplace violence is more prevalent.

Furthermore, the use of rapid assessment and tracking systems for patients and caregivers that highlight high‐risk behaviours could be valuable tools to incorporate into the clinical documentation of emergency departments. Including this information in patient records would facilitate the transfer of information among healthcare professionals, ensuring data traceability and improving continuity of care. In the context of workplace violence prevention, these tools offer significant potential to enhance both proactive and reactive strategies for managing violent incidents. However, despite their promise, more research is needed to further evaluate the long‐term effectiveness of these tools in diverse healthcare settings, particularly in terms of their integration into routine practices (Sammut et al. 2023). The validation of assessment tools is critical for early identification of individuals at risk of experiencing WPV. Validated tools could allow for reliable identification of risk factors, which can then be integrated into clinical documentation to improve tracking and ensure interventions are appropriately personalised (Kumari et al. 2021).

Alongside validated assessment tools, simulation‐based training plays a vital role in preparing healthcare professionals for potential violent encounters, offering a safe and controlled environment in which individuals can practice their responses to aggression or conflict. In fact, it was previously highlighted how the early recognition of the violent event is a protective factor (Bagnasco et al. 2024).

Moreover, a “violence prevention bundle” that grouped several interventions was successfully implemented and tested to enhance safety in healthcare settings (Mallett‐Smith et al. 2023, 341). Based on the results of this study and other literature findings, developing a specific bundle for workplace violence prevention would be valuable. This could include risk assessment tools, training components, and structured interventions for handling workplace violence that could improve safety in healthcare environments. Such a bundle would coordinate the use of validated tools for assessing risk, simulation‐based training for skill development, and evidence‐based interventions for managing violent incidents, ultimately creating a safer and more supportive workplace.

Lastly, despite evidence in the literature (Hou et al. 2024) highlighting space as a risk factor for workplace violence due to elements such as overcrowding and high ambient temperatures, our study did not identify any interventions aimed at modifying spatial environments. Future research should investigate how environmental modifications can be integrated into comprehensive workplace violence prevention strategies.

It may also be valuable to briefly discuss aspects commonly associated with interventions for the prevention or management of workplace violence (WPV) that did not emerge from the findings of this scoping review—such as artificial intelligence (AI). Specifically, AI could support the development of more personalised and adaptive training programs and enhance simulation‐based learning through advanced data analytics (Kavanagh et al. 2024). The absence of AI‐based approaches among the included studies highlights a gap in the current literature that could be addressed by future research, particularly in the development of innovative tools for training and risk assessment in Emergency Department settings.

## 6 Strengths and Limitations

This study offers valuable insights into interventions aimed at preventing violence perpetrated by patients and visitors against nurses in Emergency Departments, based on a comprehensive scoping review of 43 interventions implemented in various countries. Strategies are categorised by type—organisational, environmental, and individual‐focused—as well as by timing in relation to the violent event: before, during, or after. Interventions are also classified according to their target, including victims, perpetrators, and care settings. This structured approach provides a holistic understanding of global prevention efforts. The rigorous data extraction process, guided by established frameworks such as the Cochrane Review and WHO guidelines, enhances the reliability and relevance of the findings.

Despite these strengths, the study has several limitations. First, we acknowledge that restricting the search to PubMed and CINAHL may have led to the exclusion of relevant studies, including grey literature. However, this decision was based on the broad and complementary coverage of these databases in the fields of biomedicine and nursing, which are central to the scope of this review. Moreover, the focus on English‐language studies and the predominance of research conducted in the United States may have excluded valuable perspectives from non‐English‐speaking and non‐Western contexts, potentially limiting the global generalizability of the findings.

Second, the variation in study designs and intervention methodologies presents challenges in directly comparing the effectiveness of interventions. While the inclusion of both observational and experimental studies strengthens the findings, the lack of long‐term follow‐up data on the sustainability of interventions is a notable gap. Finally, while the study highlights the importance of organisational and individual strategies, interventions targeting environmental factors, particularly spatial elements such as overcrowding and high ambient temperatures, were underrepresented, despite their recognised role in exacerbating workplace violence. Overall, while the study provides a comprehensive overview of current interventions, further research is needed to evaluate the long‐term effectiveness of these strategies and explore underrepresented aspects, such as environmental interventions and the integration of advanced technologies like artificial intelligence.

## 7 Conclusions

This scoping review highlights several key strategies to address workplace violence in emergency departments. Simulation‐based training emerges as one of the most effective interventions, as it enhances healthcare workers' ability to recognise and manage violent incidents through experiential learning and reflective debriefing. The validation and integration of assessment tools into clinical practice represent another critical approach, allowing for early identification of individuals at risk and improving targeted interventions. Additionally, the development of comprehensive preventive bundles—integrating risk assessment, training, and structured interventions—could offer a more coordinated and evidence‐based approach to workplace violence prevention. Finally, despite the recognition of environmental factors, there is a notable gap in interventions addressing spatial modifications. Future research should explore this dimension as part of a holistic strategy to enhance safety in healthcare settings.

## Author Contributions

**Mariarosaria Gammone:** conceptualization, methodology, validation, formal analysis, investigation, data curation, validation, writing – original draft, visualisation. **Daniela Cattani:** conceptualization, methodology, validation, formal analysis, investigation, data curation, validation, writing – original draft, visualisation. **Martina Barbieri:** conceptualization, methodology, validation, formal analysis, investigation, data curation, validation, writing – original draft, visualisation. **Andrea Moro:** conceptualization, methodology, validation, formal analysis, investigation, data curation, validation, writing – original draft, visualisation. **Milko Zanini:** conceptualization, supervision. **Gianluca Catania:** conceptualization, supervision. **Loredana Sasso:** conceptualization, supervision. **Fiona Timmins:** conceptualization, methodology, review and editing, supervision. **Annamaria Bagnasco:** conceptualization, supervision.

## Conflicts of Interest

The authors declare no conflicts of interest.

## Data Availability

The data that support the findings of this study are available from the corresponding author upon reasonable request.
